# Rapid mapping of polarization switching through complete information acquisition

**DOI:** 10.1038/ncomms13290

**Published:** 2016-12-02

**Authors:** Suhas Somnath, Alex Belianinov, Sergei V. Kalinin, Stephen Jesse

**Affiliations:** 1 The Institute for Functional Imaging of Materials and The Center for Nanophase Materials Sciences, Oak Ridge National Laboratory, 1 Bethel Valley Road, Mail Stop 6487, Oak Ridge, Tennessee 37831, USA

## Abstract

Polarization switching in ferroelectric and multiferroic materials underpins a broad range of current and emergent applications, ranging from random access memories to field-effect transistors, and tunnelling devices. Switching in these materials is exquisitely sensitive to local defects and microstructure on the nanometre scale, necessitating spatially resolved high-resolution studies of these phenomena. Classical piezoresponse force microscopy and spectroscopy, although providing necessary spatial resolution, are fundamentally limited in data acquisition rates and energy resolution. This limitation stems from their two-tiered measurement protocol that combines slow (∼1 s) switching and fast (∼10 kHz–1 MHz) detection waveforms. Here we develop an approach for rapid probing of ferroelectric switching using direct strain detection of material response to probe bias. This approach, facilitated by high-sensitivity electronics and adaptive filtering, enables spectroscopic imaging at a rate 3,504 times faster the current state of the art, achieving high-veracity imaging of polarization dynamics in complex microstructures.

## Abstract (web summary)

Resolution of classical piezoresponse force microscopy is limited in data acquisition rates and energy scales. Here, Somnath *et al*. report an approach for rapid probing of ferroelectric switching using direct strain detection of material response to probe bias, enabling spectroscopic imaging at a rate of 3,504 times faster the current state of the art.

## Graphical abstract

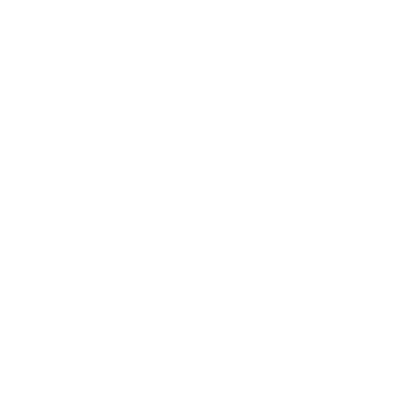

Polarization switching in ferroelectric and multiferroic materials underpins a number of applications ranging from non-volatile memories^1^, tunnelling barriers^2^, field-effect transistors^3^, race-track memory^4^, domain wall^5^ and magneto-electric devices^6^. Switching mechanisms are sensitively affected by local and extended defects that serve as nucleation centres for ferroelectric domains, and pinning sites for moving domain walls^7^,^8^. Consequently, the understanding of local switching mechanisms is paramount for these applications. Furthermore, predictive atomistic models of ferroelectric switching are possible only once information on local switching and defect types is available.

These considerations stimulated the development of scanning probe microscopy (SPM) techniques such as piezoresponse force microscopy (PFM) to probe local switching mechanisms^9^,^10^,^11^. In PFM, a biased nanoscale tip localizes the electric field within a nanometre-scale volume^12^,^13^. The electromechanical response to high-frequency electrical excitation (probing bias) detected via cantilever deflection provides information on the polarization state of the sample under the tip, constituting the imaging mode of PFM. Concurrent application of a bipolar triangular waveform (switching bias) can change polarization below the tip. Measured bias-dependent response constitutes the spectroscopic mode of PFM^14^,^15^, in which measured local hysteresis loop yields information on local polarization dynamics^16^,^17^. Finally, in the spectroscopic imaging mode of PFM, hysteresis loops are measured over a spatial grid yielding three-dimensional data sets. The hysteresis loops can be analysed to yield the parameter maps such as work of switching, nucleation, coercive bias and so on, describing the variability of polarization dynamics in materials^18^. This approach has been used to visualize phenomena including effects of dislocations^19^, grain boundaries^20^, charge screening^21^, ferroelectric^22^ and ferroelastic walls^7^,^23^, electrodes^24^ on polarization switching, polarization dynamics in ferroelectric nanostructures^25^,^26^, disordered ferroelectrics^27^ and novel ferroelectric materials^28^. Recent discoveries of static and hysteretic electromechanical responses in a broad variety of materials from non-ferroelectric oxides^29^ to energy storage^30^ and conversion^31^ materials to biomolecular and biological systems^32^,^33^ further fuel the interest in these techniques.

Despite the rapidly growing number of applications of PFM spectroscopy and imaging, the data acquisition rates and hence quality and veracity of data are severely limited by the nature of measurement process. The spatial and voltage resolution of a two-stage measurements are controlled by the interplay of several factors. First, the duration necessary for detecting differential signals (∼4 ms per voltage step for band excitation^34^ and ∼1 ms for lock-in detection, for example) limits the theoretical duration to ∼43 min for acquiring a 100 × 100 pixel image with 64 voltage steps. Although faster pixel times have been achieved in certain measurements^35^, we note that such techniques are typically limited to imaging^35^,^36^ or are single location spectroscopy techniques^37^. However, instrument communication, file output and controlling the tip movement practically sets the duration to ∼8 h for the same scan. Increasing the spatial or voltage resolutions dramatically increases the measurement time, which increases sample drift and introduces imaging instabilities. Consequently, this limits high-resolution, spatially dense PFM spectroscopic imaging to highly stable instruments. Second, good signal-to-noise ratios necessitates higher probing voltages, which in turn can affect the switching process^18^. Finally, the inertial stiffening of the cantilever at high frequencies^38^,^39^,^40^ and the photodiode bandwidth limit the probing frequency and in turn switching bias frequency.

Here we develop an approach for rapid probing of ferroelectric switching using direct strain detection of the material response to probe bias. This approach enables spectroscopic imaging 3,504 times faster than the current state-of-art techniques by combining full data acquisition^41^,^42^ and adaptive filtering. As such, this technique enables fast, pixel dense, high-resolution spectroscopic imaging of polarization dynamics in complex microstructures. We compare this technique with the current state-of-art and illustrate new methods to analyse data from this technique. By rapid acquisition of a large number of hysteretic loops on very dense grids, this technique will enable significant insight into nanoscale polarization dynamics and phenomena such as polarization fatigue or local wall displacements that remain difficult to study at the desired spatial and temporal scales, and are crucial for integration of ferroelectric nanostructures in future electronic devices.

## Results

### Measurement technique

**Figure 1:**
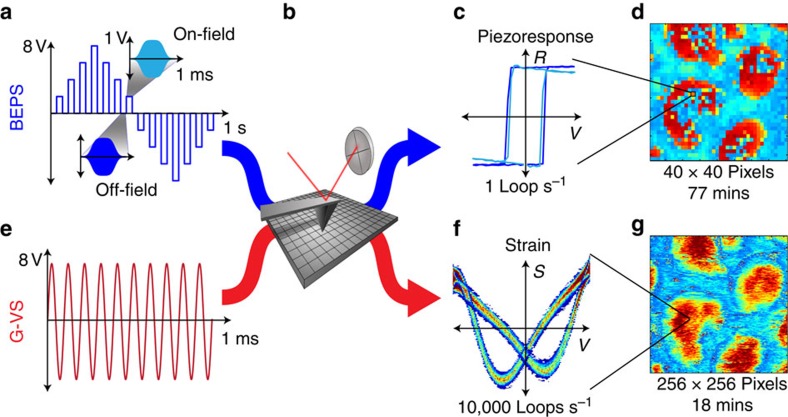
Technique comparison between G-VS and the current state-of-art BEPS. (**a**,**b**,**e**) In both techniques, the cantilever tip is excited with a waveform and the sample response is recorded through the cantilever deflection. (**a**,**c**) In BEPS, a slow bipolar triangular wave induces ferroelectric switching, while the piezoresponse is measured by exciting the cantilever with a narrow band around its contact resonance. (**e**) In G-VS, the tip is excited with a high-frequency, high-amplitude sinusoidal signal. (**f**) The cantilever response is intelligently filtered to reveal multiple strain loops. (**c**,**d**) Although BEPS is limited to ∼0.1–1 switching cycle per second and acquired over a sparse spatial grid, (**f**,**g**) G-VS results in 10^4^−10^7^ switching cycles per second over a dense spatial grid.

Figure 1 outlines the methodology for polarization switching using general-mode voltage spectroscopy (G-VS), as compared with band-excitation polarization switching (BEPS), which is the current state-of-art. In G-VS, the atomic force microscope (AFM) cantilever is excited with a high-frequency sinusoidal waveform, with amplitude that exceeds the coercive bias of the sample. The cantilever displacement is measured directly, yielding local strain hysteresis loops. BEPS excites the tip with a small amplitude chirp signal centred at the cantilever resonance frequency, whereas the DC offset is modulated by a bipolar triangular waveform with the amplitude slowly traversing the coercive bias. G-VS effectively measures bias-induced strain, whereas BEPS measures the bias-dependent piezoelectric coefficient. Although BEPS operates at about one switching cycle per second, limited by the bipolar triangular waveform, G-VS waveforms result in several tens to thousands of polarization switching cycles, limited only by the frequency (1 kHz–1 MHz) and duration (1–10 ms) of the driving waveform. In BEPS, the switching rate is determined by the number and the size of the voltage steps per cycle. Hence, BEPS has a trade-off between the switching rate and the voltage resolution, whereas G-VS does not. These factors allow G-VS to achieve very fast polarization switching rates without sacrificing voltage resolution.

The fundamental differences in signal generation between G-VS and BEPS translate to different approaches in processing data. Traditional AFM optics record the electromechanical response of the sample via the vertical cantilever deflection. Although BEPS only retains the response from the frequency bands that were excited, G-VS stores the complete cantilever response for postprocessing. The availability of the complete response signal provides true, impartial information on the cantilever response that includes multiple vibrational modes and the harmonics of the drive frequency^41^. In contrast, the traditional heterodyne methodology of PFM with a single excitation frequency (S-PFM) restricts information to monitoring a single frequency by using a lock-in amplifier and averaging the signal to a single value^41^,^43^. Finally, in BEPS the data are fitted to a simple harmonic oscillator model to the piezoresponse^34^,^44^, as dictated by the nature of capturing a single resonance band of the cantilever, whereas G-VS allows a wider model selection with multiple interacting resonances and harmonics^45^.

**Figure 2:**
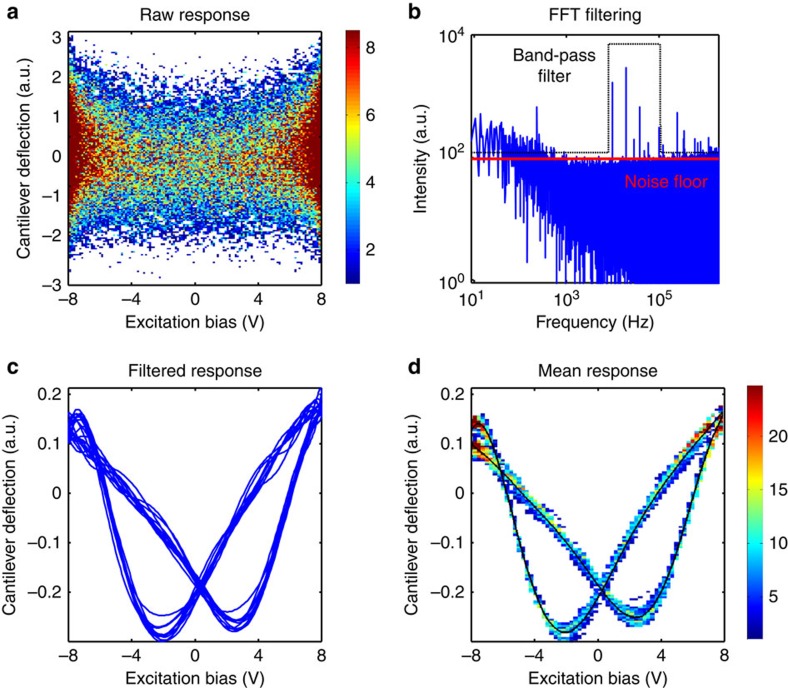
Methodology for filtering the G-VS data. (**a**) 2D histogram of the raw cantilever deflection signal as a function of the tip excitation bias. (**b**) The raw signal is filtered in the frequency domain by discarding the signal below a calculated noise floor and by applying a band-pass filter to reject low and high frequency noise. (**c**) The filtered deflection signal showing strain loops. (**d**) The filtered signal is used to populate a 2D histogram and a mean strain loop (inner, black line) is constructed from the mean response at each voltage step.

Following data acquisition, G-VS uses data-driven postprocessing to reveal multiple strain loops that are indicative of polarization switching, as illustrated in Fig. 2. As the duration of the G-VS excitation signal is comparable to the time per pixel (1–10 ms), G-VS can be integrated into a continuous scan as opposed to the grid approach used in BEPS. The continuous, smooth motion of the tip minimizes drift and implements spectroscopy at imaging speeds. Although the simplest case of single-frequency G-VS excitation is discussed here, the same principles can be extended to dual frequency^46^, bimodal^47^, band excitation^34^ or any other excitation waveform.

As is the raw cantilever response, shown in Fig. 2a, seems unintelligible and is therefore processed to extract material strain information. The signal is transferred into the frequency domain using a fast Fourier transform (Fig. 2b). The quality of the data is used to calculate an appropriate noise-floor dynamically, as opposed to using an arbitrary, a priori value, as shown by the red line in Fig. 2b. Subsequently, a band-pass filter, shown as the dotted black line in Fig. 2b, is applied to reject low- and high-frequency noise. The band-pass filter retains the response from the first 11 harmonics of the excitation frequency. Contributions from known noise bands of the microscope are also rejected from the signal and the signal below the calculated noise floor is rejected. Finally, the data are returned into real-space via an inverse fast Fourier transform. Figure 2c illustrates the filtered signal containing multiple strain loops, corresponding to the multiple switching cycles. The drive and response signals are rearranged based on the slope of the drive signal and a two-dimensional (2D) histogram is constructed using the rearranged signals. Average strain loops are constructed from the centre of mass at each voltage step in the histogram, with the results shown in Fig. 2d. The postprocessing steps are necessary for processing G-VS data, as the tip-sample response is typically distributed over several frequency bands and the frequency bands of the tip-sample response can shift, precluding digital lock-in based processing^48^. Additional measurement channels such as the lateral deflection can be acquired and processed similarly in parallel.

**Figure 3:**
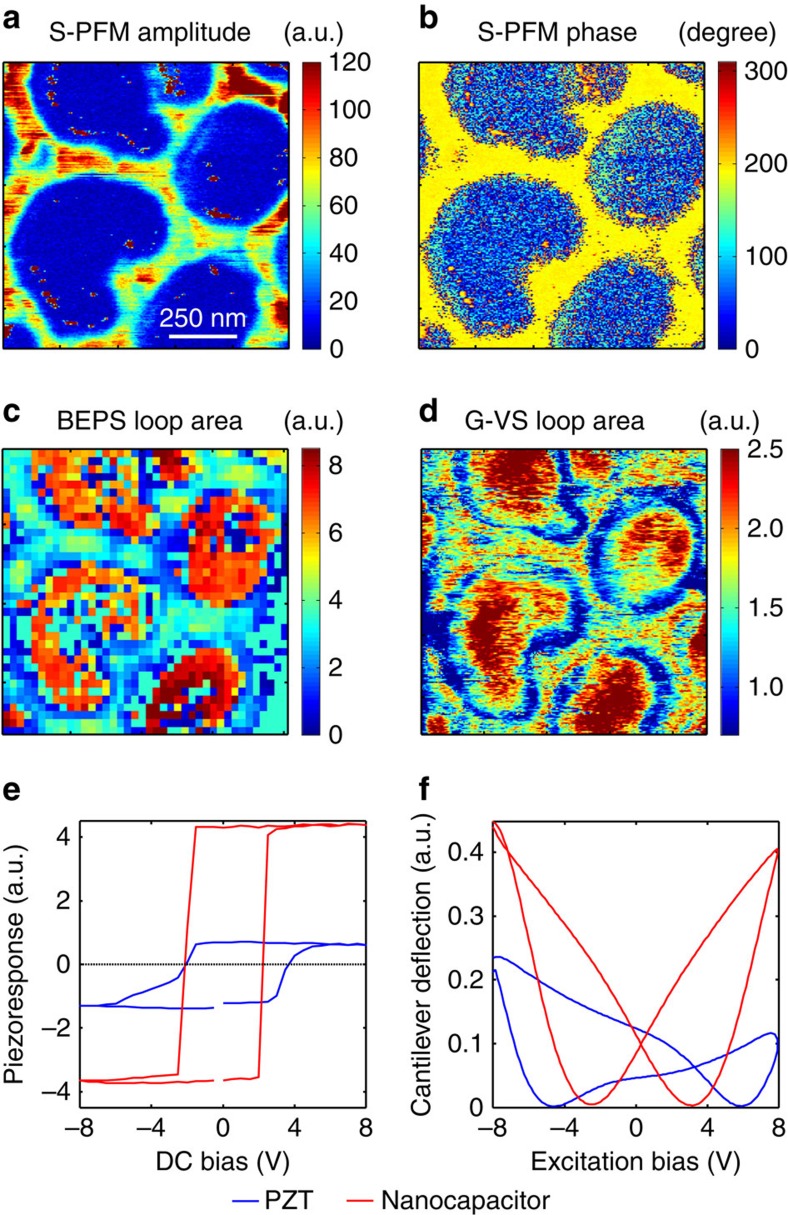
Information content in different PFM imaging and spectroscopic techniques. Images of a PZT-nanocapacitor sample obtained from S-PFM, BEPS and G-VS. S-PFM (**a**) amplitude and (**b**) phase maps. (**c**) Total piezoresponse loop area from BEPS. (**d**) Total strain loop area from G-VS. (**e**,**f**) Average response on the nanocapacitors and PZT areas. (**e**) Piezoresponse from BEPS data. Piezoresponse (PR) is defined as PR(*V*)=*A*(*V*)*cos(*ϕ*(*V*)) where *A* and *ϕ* are the amplitude and phase as a function of the applied DC bias, *V*. (**f**) Mean strain loops from G-VS data.

Here we realize G-VS for single-frequency sinusoidal excitation on a sample with nanocapacitors. The sample consists of a [001] Pb(Zr_0.2_Ti_0.8_)O_3_ (PZT) film sandwiched between a SrRuO_3_ electrode on a SrTiO_3_ substrate, with top electrodes that are Au/Cu discs 300 nm in diameter and 20 nm tall^49^. The 1 × 1 μm^2^ areas were investigated by S-PFM, BEPS and G-VS. Additional details of the experimental setup can be found in the Methods section. Figure 3 compares the information provided by the different PFM modalities. The S-PFM amplitude and phase images in Fig. 3a,b contain 256 × 256 pixels and were acquired in ∼18 min. Although S-PFM is fast, it does not provide quantitative information on polarization switching, cantilever-sample interactions or frequency crosstalk^50^. The BEPS measurement in Fig. 3c was acquired on a 40 × 40 pixel grid in 77 min. Each pixel contain two polarization-switching cycles at 64 voltage steps per cycle. The G-VS map in Fig. 3d is a 256 × 256 pixel map acquired in 18 min. Each pixel contains 40 strain loops in a 16,384 point array.

All SPM imaging and spectroscopy measurements suffer from a tradeoff between the spatial resolution and the imaging area. However, the poor speed of BEPS results in a very significant tradeoff compared with G-VS or S-PFM. The fast speed of G-VS allows a dense grid of measurements over areas spanning several micrometres, while retaining sub-50 nm spatial resolution. Furthermore, the availability of the continuous data stream allows multi-resolution imaging in the fast scanning direction^41^,^51^. In other words, the G-VS data can be converted from a 256 × 256 image with 40 loops per pixel to a 256 × 2,560 image with 4 loops per pixel, thereby allowing the adjustment of the spatial resolution after the data were acquired.

Figure 3e,f shows the spatially averaged BEPS and G-VS response from the nanocapacitors and the bare PZT film. The BEPS and G-VS data show distinctly different responses for the PZT and nanocapacitors and the coercive biases calculated from both techniques are in good agreement. BEPS would take 3,504 × longer than G-VS to provide a comparable spectroscopic image with 256 × 256 pixels, where each pixel contains 40 loops. Overall, G-VS can be as fast as S-PFM imaging and 3,504 times faster than BEPS, providing information that is complementary to both techniques. Furthermore, the amplitude of the G-VS excitation waveform could be modulated such that the material response can be rapidly studied for a variety of excitation biases without having to re-do the experiment for different excitation bias amplitudes. Supplementary Fig. 1 shows the sample response to such time-varying excitation bias. This approach is substantially faster than the BEPS equivalent and could potentially be used to construct Preisach density maps rapidly^52^.

### Data mining

**Figure 4:**
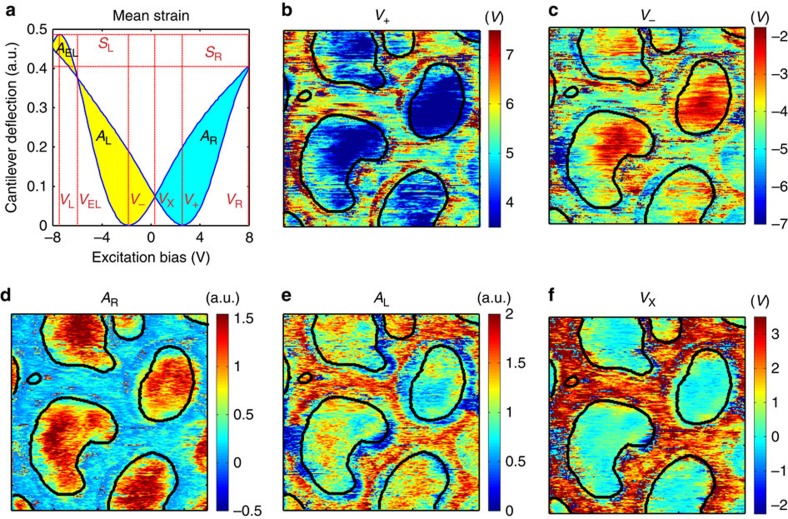
Analysis and spatial variation of the strain loop shape. (**a**) Derivation of metrics from the mean strain loop. Table 1 lists the abbreviations and description of all metrics. Spatial maps of (**b**) forward coercive bias, *V*_+_, (**c**) reverse coercive bias, *V*_−_, (**d**) area within right wing, *A*_R_, (**e**) area within left wing, *A*_L_, and (**f**) cross-over bias, *V*_X_. The black outline in the spatial maps indicates the edges of the nanocapacitors.

Material specific properties can be extracted from the shapes of the G-VS strain loops. Here, the slope and the offset in the mean strain loops are removed before analysis. Figure 4a shows how metrics such as the forward and reverse coercive bias, areas within the left and right wings of the strain loops, are measured in the averaged strain loops. Figure 4b–f shows the spatial distribution of some loop metrics. Spatial maps of all G-VS and BEPS loop metrics are shown in Supplementary Figs 2 and 3, respectively. Table 1 lists the metrics extracted from the G-VS loop shapes. Certain parameters such as *A*_R_, *V*_X_ show clear contrasts between the PZT and nanocapacitors, while other metrics such as the *V*_*−*_ show poorer contrast between the PZT film and the capacitor.

The similarity in the spatially distributions of loop metrics can be quantified through the cross-correlation coefficient *r* defined in equation 1 as:


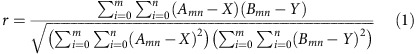


**Figure 5:**
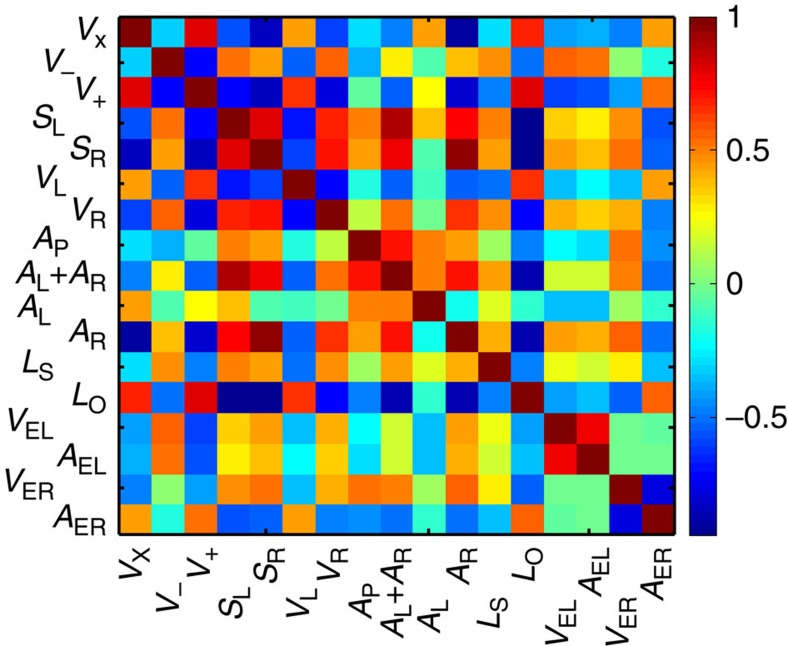
Mining the data derived from the strain loop shape. 2D self-correlation functions of the spatial maps of different loop metrics.

Here *A* and *B* are 2D input images and *X* and *Y* are the 2D mean values of *A* and *B*, respectively. *r*-values range from −1 (perfectly anti-correlated) to 1 (perfectly correlated) and a value of 0 suggests that the images are independent. Figure 5 shows the cross-correlation between spatial maps of 16 loop metrics. These correlation coefficients show how certain geometric loop parameters match with other conventional metrics such as the coercive biases, either due to material-specific correlations or interdependence between defined variables. For example, the correlation coefficients between *A*_R_ and *L*_O_, *A*_L_*+A*_R_, *V*_*+*_ and *V*_X_ are strong, whereas that between *A*_L_ and *S*_R_, *V*_L_, *V*_R_ and *L*_O_ are weak.

Further mining of the G-VS data is performed through multivariate statistical methods, such as principal component analysis (PCA), *k*-means clustering and Bayesian linear unmixing (BLU)^53^,^54^,^55^. PCA separates data into orthogonal components that are arranged in descending order by variance^56^. Results of PCA applied to the filtered G-VS data are shown in Supplementary Figs 4–6. The filtered G-VS data can also be de-correlated and compressed using PCA. Supplementary Fig. 5 shows that the first 18 of the 16,384 components contain the majority of the physically relevant information and the first 64 components are sufficient to accurately reconstruct the filtered G-VS data. Thus, the typical G-VS raw data file, of size 2 GB, can be stored permanently as an 8 MB file without much loss in information^41^,^42^. Supplementary Fig. 7 shows the cross-correlation between the sample topography, spatial maps of G-VS and BEPS loop metrics, and the G-VS PCA loadings maps.

**Figure 6:**
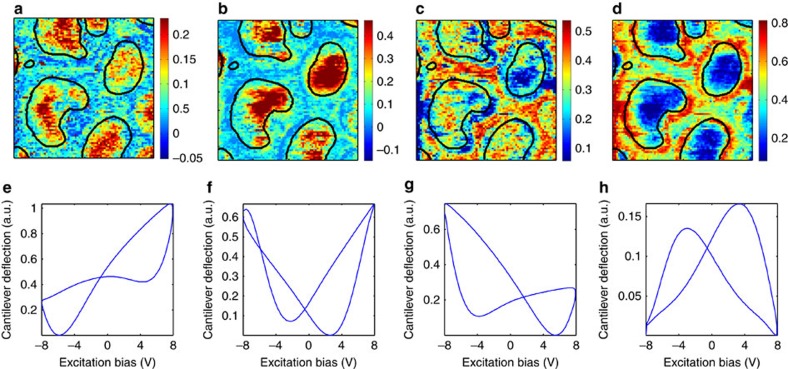
Bayesian Linear Unmixing of G-VS strain loops into four components. (**a**–**d**) Spatial maps and (**e**–**h**) corresponding strain loops for each of the four endmembers. The edges of the nanocapacitors (black lines) are overlaid on the spatial maps.

BLU separates observations into a linear combination of position-independent endmembers with relative abundances that are corrupted by additive Gaussian noise^57^. The *k*-means algorithm attempts to classify the data into *k* clusters wherein each pixel belongs to the cluster with the most similar response^58^. As the majority of the statistically relevant information in the filtered G-VS data is contained within the first four principal components, *k*-means and BLU were configured to identify four clusters or endmembers. Figure 6 shows the results of BLU on the mean G-VS strain loops, identifying regions with defined switching dynamics. Supplementary Fig. 8 shows the results of *k*-means clustering on the mean G-VS strain loops. *k*-means also separates the response from the bare PZT film, centre of the nanocapacitors, edges of the nanocapacitors and other sections having very weak response.

## Discussion

An important step in processing the strain loops is the compensation of the phase offset between the excitation bias and the measured deflection, as the phase can significantly affect the shape of the hysteresis loop. For a purely linear, non-hysteretic response, this phase offset can change loops from closed to ellipsoid in shape. As the SPM system has finite frequency dispersion of the response, direct calibration of the phase is non-trivial. The phase offset should be chosen such that the resultant loop shape is closest to the classical shape of strain loops under switching conditions for ferroelectrics. Before performing the polarization switching G-VS experiment described in this study, the same area was imaged with the G-VS amplitude well below the coercive bias −1 V in this case. Applying the filtering routines described in Figure 2 revealed oval-shaped loops at every pixel. The phase offset was calculated as the relative rotation between the input and output waveforms necessary to minimize the area within the loop. The difference between the shapes of the phase-compensated and uncompensated loops was negligible. In our experiments, the phase offset and the effects of the phase correction on the data set were negligible.

High-speed switching and complete data capture in G-VS enables a number of material science investigations that have traditionally been impractical or challenging. G-VS can be used for rapid studies on the fatigue behaviour in novel materials. In this study, G-VS is implemented by exciting the cantilever well below the first resonance frequency where the transfer function between the measured cantilever deflection and material strain is linear. Operating the cantilever near a resonance mode significantly improves the signal-to-noise ratio due to the inherent amplification nature of the cantilever. However, at resonance conditions, complex cantilever dynamics models are necessary to relate the deflection with the material strain, which is beyond the scope of the current work. The integration of cantilever dynamics models into the G-VS data analysis will enable investigations of material response at arbitrary frequencies. G-VS can also provide valuable insights about the relative contributions of ionic and ferroelectric phenomena in the material response of certain samples, for example, Ca-doped BiFeO_3_ (ref. 59) and LaAlO_3_-SrTiO_3_ (ref. 60) structures. The dynamics of domain wall motion and frequency dependence of nucleation bias can also be investigated. The enhanced spatial and temporal resolution of the technique should also allow significant progress to be made regarding mechanisms of polarization fatigue (and perhaps tie them to the presence of mobile or static defects^19^), as well as interactions between domain walls and local nonlinearities. Furthermore, these experiments can provide a window to the links between local (nanoscale) hysteresis and macroscopic dielectric hysteresis loops, which remain poorly understood^61^ despite decades of research and often lack physical meaning for the statistical descriptors used^62^. Besides polarization switching in ferroelectrics, G-VS can be applied to other SPM methods such as Kelvin probe force microscopy^63^, force spectroscopy^34^, electrochemical strain microscopy^64^ and so on. Techniques shown in this study can potentially allow complete force-volume mapping during conventional intermittent contact mode imaging, while the cantilever is operated at resonance. Here, the response would have to be normalized by the cantilever transfer function. In comparison, commercially available rapid spectroscopy techniques, such as PeakForce Tapping mode^65^ from Bruker and Fast Force Mapping modes from Asylum Research are quasi-static modes that are limited to 10–2,000 Hz.

Here we have demonstrated a voltage spectroscopy technique that is >3,500 times faster than the current state-of-art. The improvement in the measurement speed has reduced spatial drift and improved spatial resolution, allowing large-area imaging at high resolution. We believe that this technique is preferable to traditional lock-in or band excitation techniques, as it captures the complete tip-sample interactions at the bandwidth of the detector. The capabilities and potential applications of this technique can be further expanded through software modifications and by robust modelling. Furthermore, the general principles that underpin this voltage spectroscopy approach can extended to other techniques within and beyond SPM. This technique enables reliable and thorough understanding of ferroelectric switching, which is crucial for building accurate atomistic models that will further the development of the next generation of electronic devices.

## Methods

### Cantilever and AFM configuration

Pt-Cr-coated Nanosensors Multi-75EG AFM cantilevers were used to acquire the single frequency and G-VS scan data in an Asylum Research Cypher AFM system. Single-frequency PFM images were acquired using the built-in AFM software package. Sinusoidal voltage of 1 V amplitude and frequency close to the first contact-resonance mode of the cantilever-sample system (∼350 kHz) was applied to the cantilever tip as the cantilever scanned the sample in contact mode.

### Data acquisition

National Instruments Arbitrary Waveform Generator (PXIe 5412) and Digitizer (PXIe 5122) cards were used for concurrently supplying the G-VS excitation signal and measuring the cantilever response at a sampling rate of 4 MHz. During G-VS measurements, a 10 kHz sinusoidal waveform with 8 V amplitude was applied to the tip for 4 ms at each pixel. G-VS imaging was performed in a four-pass mode (NAP mode) where the initial trace and retrace are conventional S-PFM scans performed using the traditional force-feedback method. The second trace and retrace are performed without the force feedback and G-VS is performed only during the retrace. In principle, G-VS could be implemented in the traditional two-pass scan mode with force feedback, which would halve the scan time. We implemented G-VS in the four-pass mode for easier comparison between S-PFM and G-VS data. Data are continuously recorded during the G-VS retrace; thus, the number and duration of pixels can be varied inversely during postprocessing to adjust the spatial resolution of the image.

### Data availability

All data and analysis computer code described in this study are available from the authors.

## Additional information

**How to cite this article:** Somnath, S. *et al*. Rapid mapping of polarization switching through complete information acquisition. *Nat. Commun.* **7,** 13290 doi: 10.1038/ncomms13290 (2016).

**Publisher's note:** Springer Nature remains neutral with regard to jurisdictional claims in published maps and institutional affiliations.

## Supplementary Material

Supplementary Information Supplementary Figures 1-8 and Supplementary Tables 1-2

## Figures and Tables

**Table 1:** List of G−VS loop metrics.

**Acronym**	**Loop metric**	**Acronym**	**Loop metric**
*L*_S_	Slope of the mean strain loop	*L*_O_	Offset of the mean strain loop
*V*_*+*_	Forward coercive bias	*V*_*−*_	Reverse coercive bias
*S*_L_	Maximum strain on left wing	*S*_R_	Maximum strain on right wing
*V*_L_	Excitation bias at point of maximum strain on left wing	*V*_R_	Excitation bias at point of maximum strain on right wing
*A*_L_	Area within the left wing	*A*_R_	Area within the right wing
*A*_L_*+A*_R_	Area within the left and right wings	*A*_ER_	Area within the ear of the right wing
*A*_EL_	Area within the ear of the left wing	*V*_ER_	Excitation bias at the starting point for the ear within the right wing
*V*_EL_	Excitation bias at the starting point for the ear within the left wing	*V*_X_	Excitation bias at which the left and right wings meet

Each loop metric corresponds to a geometric measurement of the mean strain loop shape, such as length or area of a component.
