# Supplementary material for: Rapid mapping of polarization switching through complete information acquisition
Source: Nat Commun. 2016 Dec 2;7:13290. doi: 10.1038/ncomms13290 (PMC5146286; doi:10.1038/ncomms13290)
Supplement: Supplementary Information — Supplementary Figures 1-8 and Supplementary Tables 1-2 [file ncomms13290-s1.pdf]

Supplementary Figures

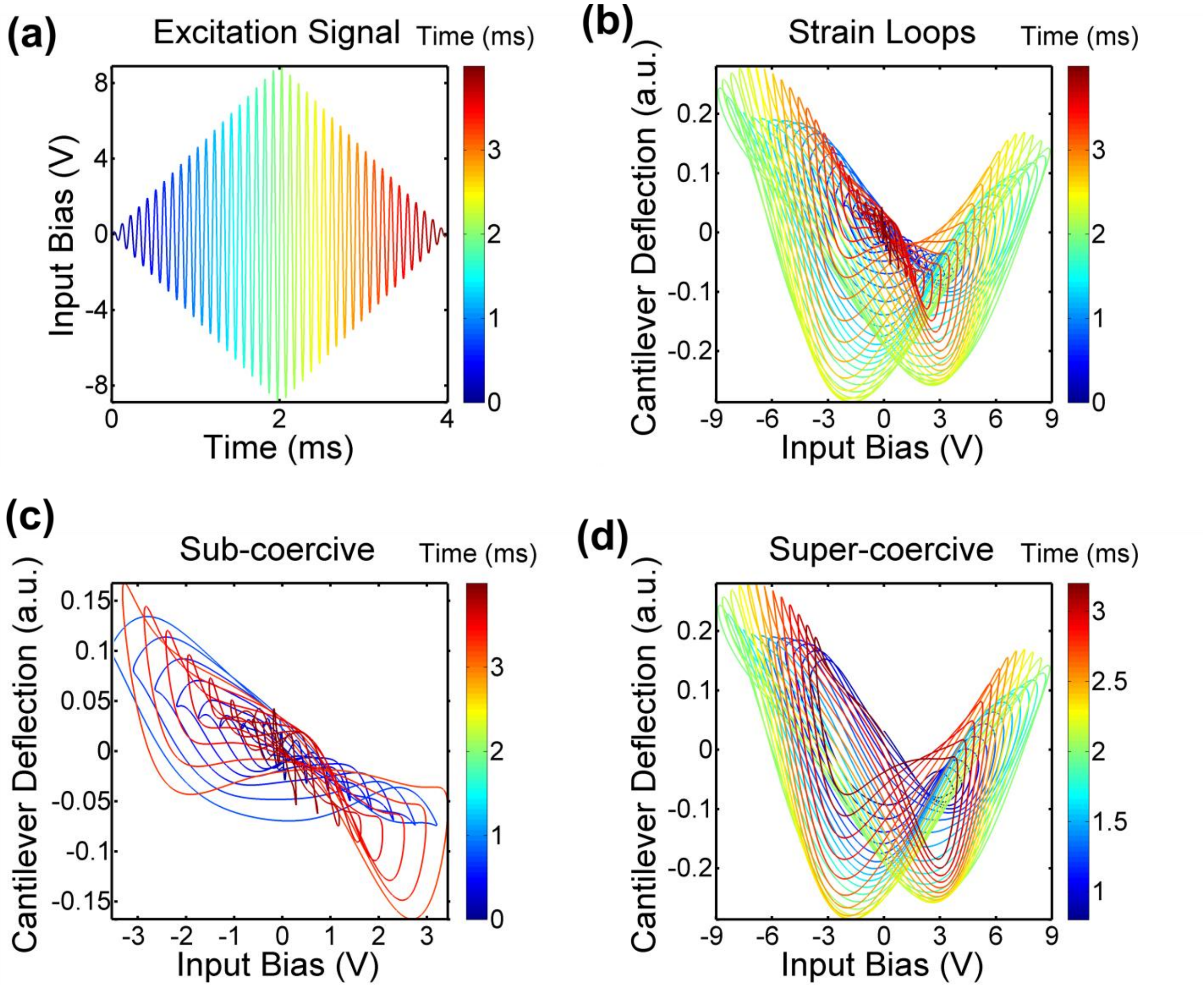

Supplementary Figure 1. Material response to time-varying excitation. (a) Excitation bias as a function of time showing the triangular envelope or modulation function. (b) Filtered cantilever deflection signal as a excitation bias showing the growth and shrinking of the butterfly strain loops. (c) Strain loops isolated for sub-coercive excitation, which do not show butterfly loops. (d) Strain loops isolated for super-coercive excitation, which show the evolution of the buttefly loops.

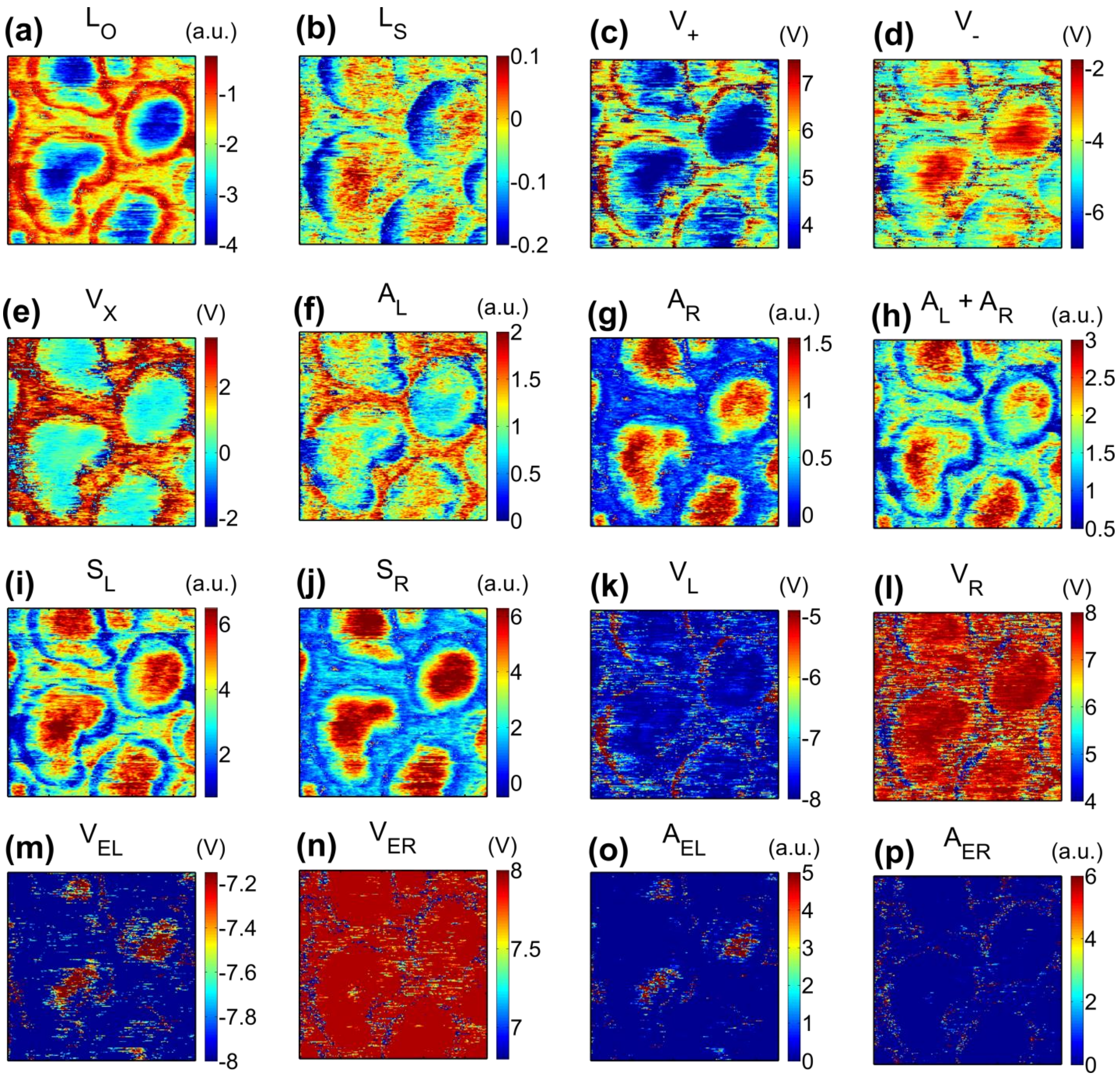

Supplementary Figure 2. Spatial variations of G-VS strain loop shapes. Some geometrical parameters show strong dependence on the material properties while others appear to be edge effects. Table 1 lists the abbreviations and descriptions of the loop shape metric.

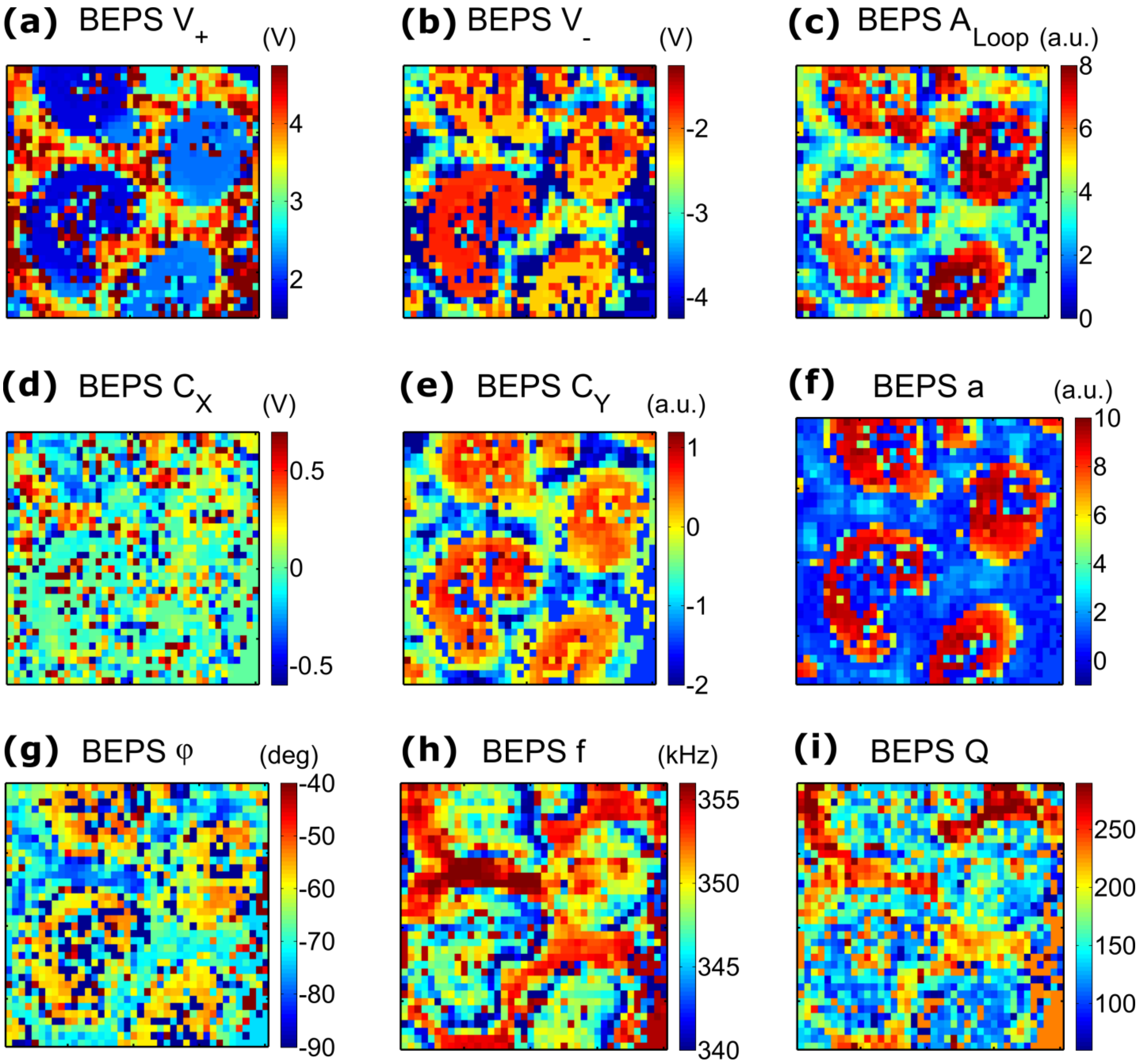

Supplementary Figure 3. Spatial maps of loop shape metrics derived from BEPS. The acronyms for these metrics are explained in supplementary table 1.

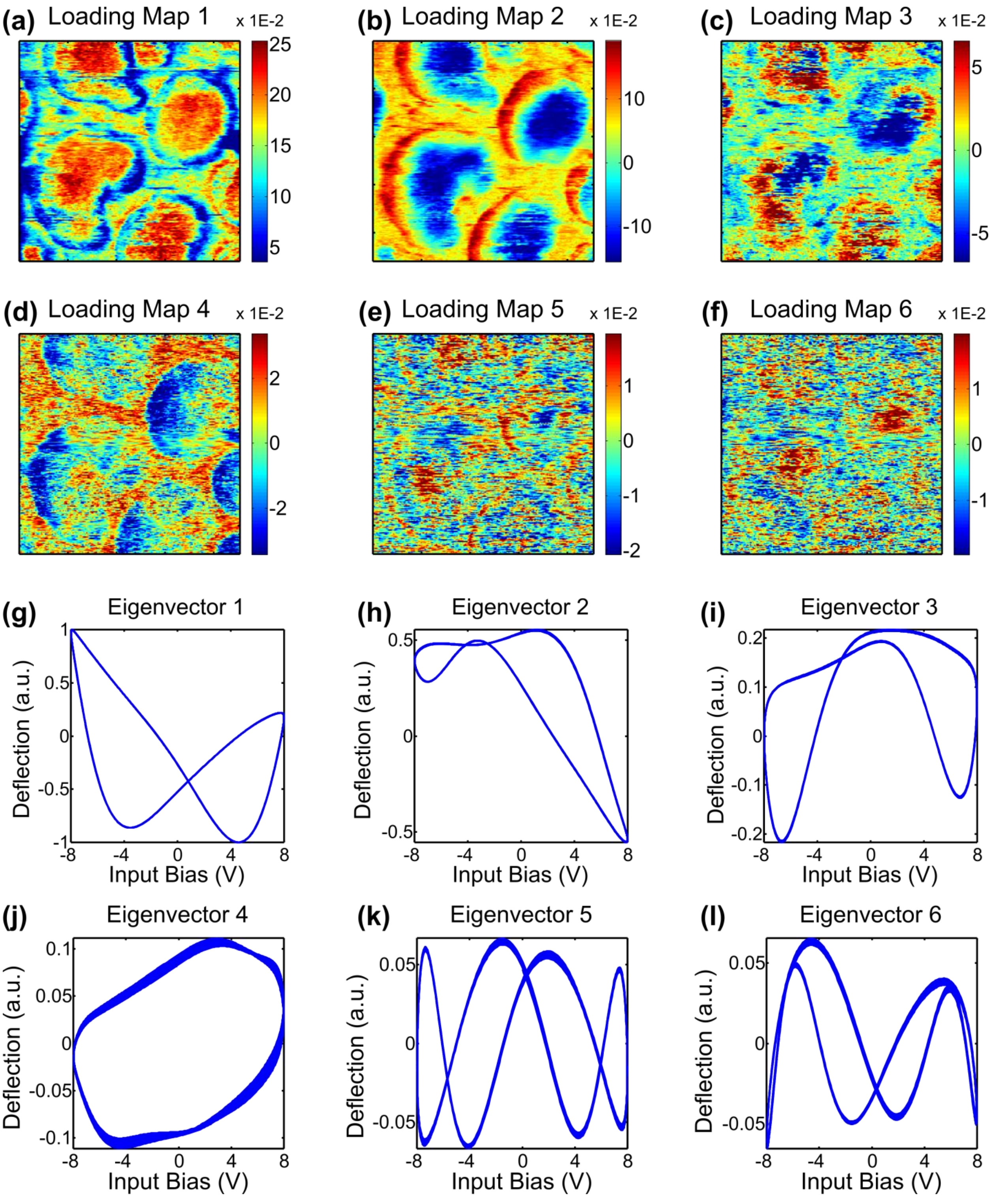

Supplementary Figure 4. **Results of PCA applied to the G-VS dataset.** The first six (a-f) loading maps and (g-l) corresponding eigenvectors plotted against the excitation bias.

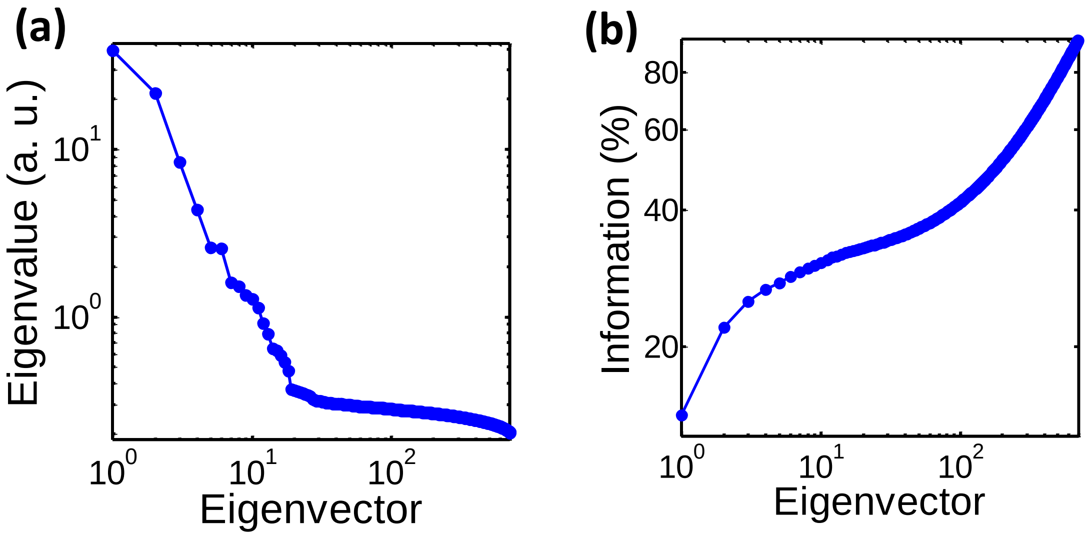

Supplementary Figure 5. **Information content in PCA results.** (a) PCA scree plot on a log-log scale. (b) Percentage of total information contained within the first few eigenvectors. The first 18 eigenvectors appear to contain the majority of the physically relevant information (36%) while the rest mainly contain noise.

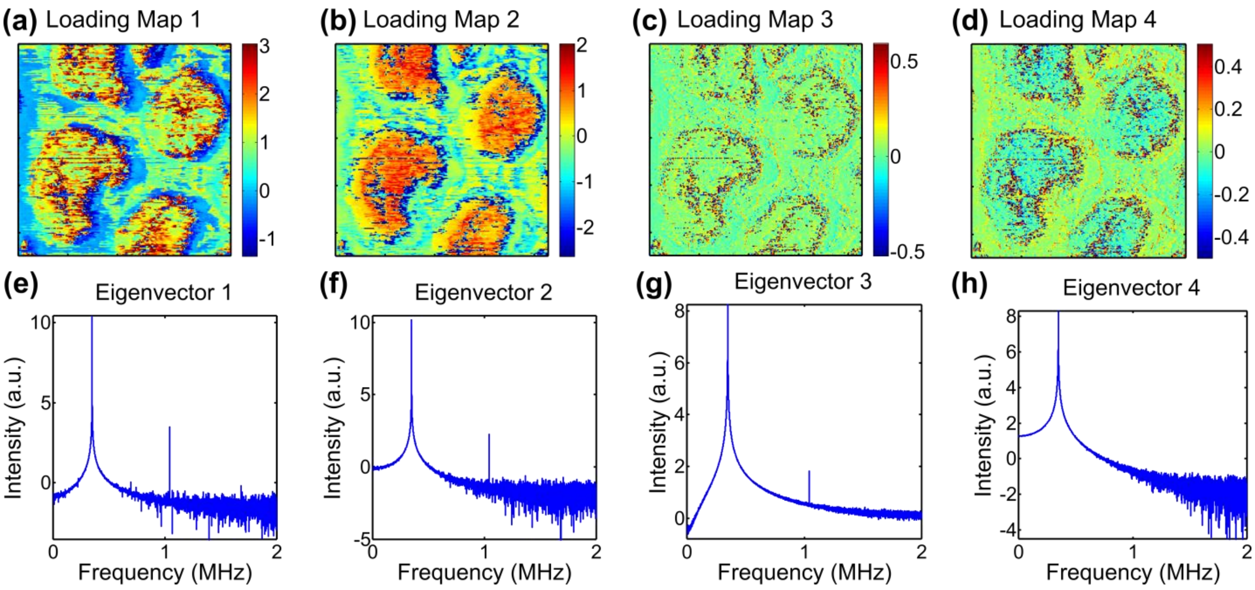

Supplementary Figure 6. **PCA of the sample response for sub-coercive excitation.** In this experiment, the sample was excited with a sine waveform at frequency 335 kHz and amplitude of 1.5 V. As expected, we do not see any strain loops. Hence, the data is represented as a function of response frequency instead of the excitation bias. The data was not filtered in any way unlike the G-VS data. The first four (a-d) loading maps and corresponding (e-h) eigenvectors in frequency space.

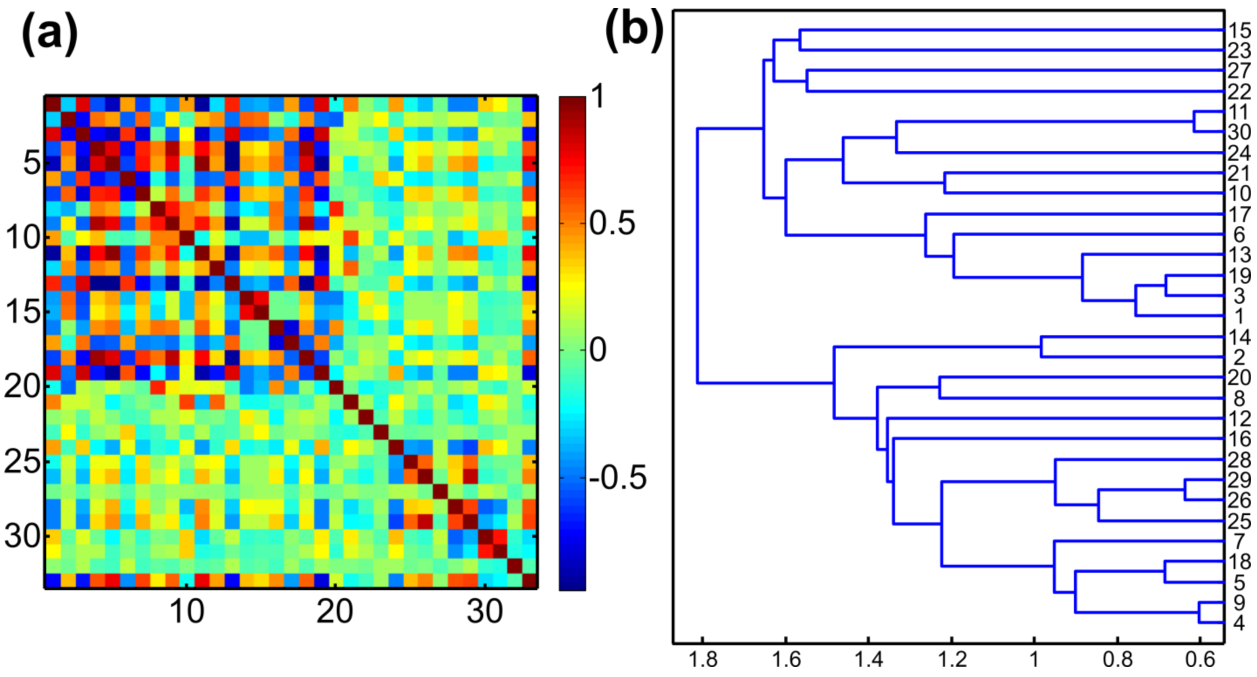

Supplementary Figure 7. **Cross correlation analysis and data mining**. (a) Autocorrelation functions between the spatial maps of the topography, G-VS loop metrics, G-VS PCA loading maps, and BEPS loop metrics. The index of the spatial maps is listed in Supplementary Table 2. (b) A dendrogram that clusters the aforementioned spatial maps based on the correlation. coefficient.

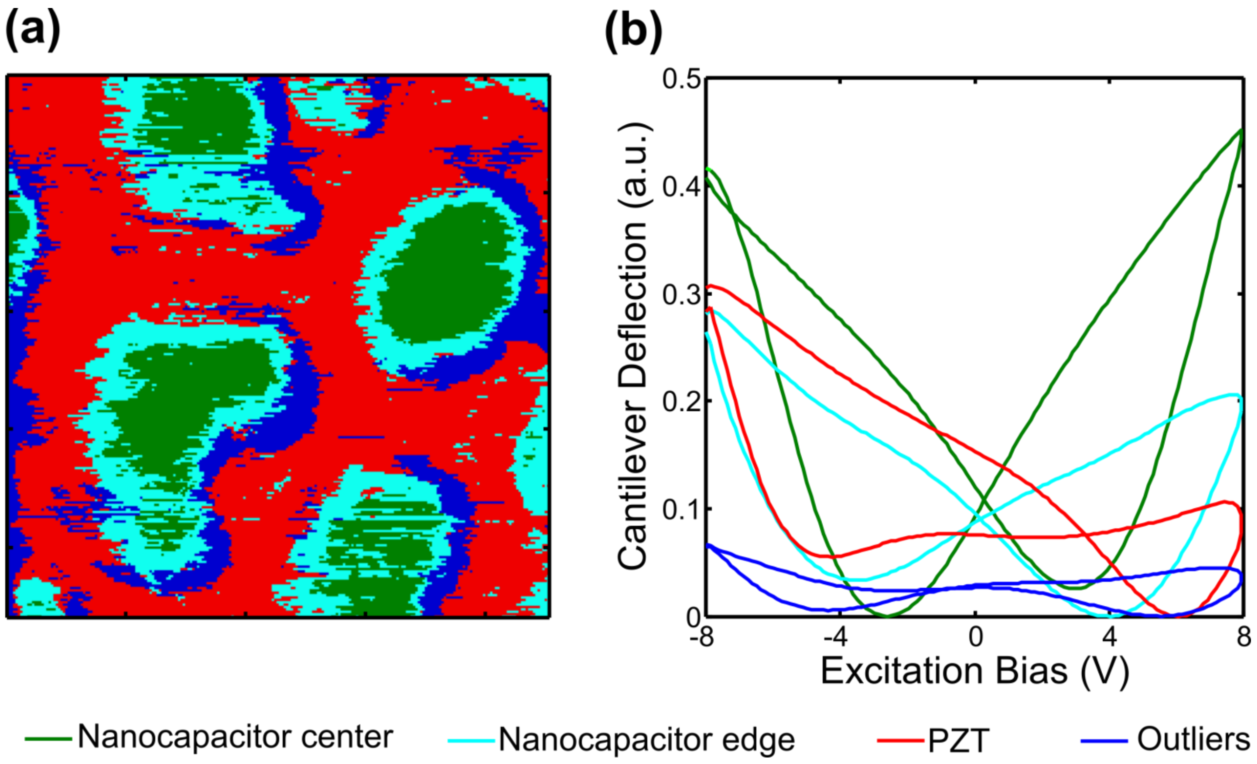

Supplementary Figure 8. **k-means clustering applied to the G-VS data**. k-means clustering identifies distinct types of response and their spatial distribution. (a) Spatial distribution of the clusters. (b) The centroid, or mean response, from each cluster. The algorithm clearly separates the centers of the nanocapacitors from the bare PZT film. The other two clusters contain response from the edges of the nanocapacitors and the bare PZT film that surrounds the nanocapacitors (NC).

Supplementary Tables

Supplementary Table 1. Descriptions of BEPS metrics. These are geometrical parameters extracted from the shape of BEPS polarization loops.

Acronym	Loop Metric	Acronym	Loop Metric
BEPS V_+	Forward Coercive bias	BEPS V_-	Reverse Coercive bias
BEPS C_X	DC Bias (x) component of the centroid of piezoresponse loop	BEPS C_Y	Piezoresponse (y) component of the centroid of piezoresponse loop
BEPS a	Amplitude	BEPS Q	Quality factor
BEPS f	Resonance frequency	BEPS φ	Phase
BEPS A_{Loop}	Area within piezoresponse loop		

Supplementary Table 2. Index of G-VS, BEPS, and topography spatial maps used in Supplementary Figure 7

Index	Map name	Index	Map name	Index	Map name
1	G-VS V_X	12	G-VS L_S	23	G-VS PCA Loading 6
2	G-VS V_-	13	G-VS L_O	24	BEPS V_+
3	G-VS V_+	14	G-VS V_{EL}	25	BEPS V_-
4	G-VS S_L	15	G-VS A_{EL}	26	BEPS A_{Loop}
5	G-VS S_R	16	G-VS V_{ER}	27	BEPS C_X
6	G-VS V_L	17	G-VS A_{ER}	28	BEPS C_Y
7	G-VS V_R	18	G-VS PCA Loading 1	29	BEPS a
8	G-VS A_P	19	G-VS PCA Loading 2	30	BEPS Q
9	G-VS A_L+A_R	20	G-VS PCA Loading 3	31	BEPS f
10	G-VS A_L	21	G-VS PCA Loading 4	32	BEPS φ
11	G-VS A_R	22	G-VS PCA Loading 5	33	Height
